# Experimental Transmission of the Yeast, *Metschnikowia bicuspidata*, in the Chinese Mitten Crab, *Eriocheir sinensis*

**DOI:** 10.3390/jof8020210

**Published:** 2022-02-21

**Authors:** Hongbo Jiang, Jie Bao, Gangnan Cao, Yuenan Xing, Chengcheng Feng, Qingbiao Hu, Xiaodong Li, Qijun Chen

**Affiliations:** Key Laboratory of Livestock Infectious Diseases in Northeast China, Ministry of Education, Shenyang Agricultural University, Shenyang 110866, China; jianghb@syau.edu.cn (H.J.); baojie@syau.edu.cn (J.B.); 2020240553@stu.syau.edu.cn (G.C.); 2007500007@syau.edu.cn (Y.X.); 2018500015@syau.edu.cn (C.F.); huqingbiao@syau.edu.cn (Q.H.); lixiaodong@syau.edu.cn (X.L.)

**Keywords:** *Metschnikowia bicuspidata*, *Eriocheir sinensis*, horizontal transmission, vertical transmission, experimental infection

## Abstract

The Chinese mitten crab, *Eriocheir* *sinensis*, is an important farmed crustacean species in China, outranking other farmed crabs in yield and economic importance. An infection called “milky disease”, caused by the yeast, *Metschnikowia* *bicuspidata*, has emerged in *E. sinensis* farms in northeast China and has caused progressive economic losses. The diseased crabs present with opaque, whitish muscles and milky hemolymph. Currently, there are no effective drugs to treat the infection. Clarifying the transmission route of *M. bicuspidata* would help to treat and prevent the disease. We investigated the effects of three different *M. bicuspidata* infection methods (feeding, immersion, and cohabitation) on *E. sinensis*. All three infection methods led to a high infection rate in healthy crabs. After 35 d, the infection rate was 76.7%, 66.7%, and 53.3% in the feeding, immersion, and cohabitation groups, respectively. Diseased crabs exhibited the typical symptom of hemolymph emulsification, with a high pathogen load of *M. bicuspidata*. The yeast was not detected in the oocytes of infected crabs. Fertilized embryos, zoea larvae, and megalopae of infected ovigerous crabs tested negative for yeast, indicating that direct transmission from mother to offspring does not occur. Our results highlight avenues for the prevention and control of this yeast.

## 1. Introduction

Crustacean culture is often accompanied by a variety of diseases, among which viral and bacterial diseases are widespread. However, studies have shown that yeast, including *Metschnikowia*, *Cryptococcus*, *Candida*, *Pichia*, *Debaryomyces*, *Torulopsis*, *Fonsecaea*, and *Exophiala*, are pathogenic to crustaceans [1,2,3,4], causing substantial economic losses to the aquaculture industry. For example, the yeast species *Candida sake*, *Pichia anomala*, *Endomyces fibuliger*, *C. famata*, and *Torulopsis mogii* are pathogenic to the freshwater prawn, *Macrobrachium rosenbergii* [5,6]. *Exophiala cancerae* and *Fonsecaea brasiliensis* are pathogenic to the mangrove land crab, *Ucides cordatus* [7], and *Metschnikowia bicuspidata* is pathogenic to the Chinese swimming crab, *Portunus trituberculatus* [8].

The Chinese mitten crab, *Eriocheir sinensis*, is an important farmed crustacean species in China, with 778, 682 tons produced in 2019 [9]. An emerging disease, which is commonly known as “milky disease”, occurred in *E. sinensis* farms in Panjin city, northeast China, in the winter of 2018 [10]. The symptoms of diseased crabs were characterized by opaque whitish muscles and milky hemolymph, inactive and anorexic behavior, and staying at the shallow end of the pond. The disease had a mortality rate of over 20% [11], and the mortality of infected crabs is rising. Thus, the disease has caused serious production and economic losses in the crab farming industry. The main pathological feature of the disease is the presence of severe myopathy, discrete necrotic lesions, and severe colonization of the pathogen in the muscles, heart, gills, and other organs. Subsequently, milky disease is of great concern to aquaculturists and researchers. Using molecular methods [11,12], the pathogen responsible for milky disease was isolated and identified as the yeast *Metschnikowia bicuspidata*.

*Metschnikowia bicuspidata* is an opportunistic pathogenic yeast that is distributed in marine and freshwater environments worldwide. Many species have been reported to host *M. bicuspidata*, including the bait organisms, *Daphnia* and *Artemia* [13,14], and more notably, aquatic animals of high economic value, such as salmon, *Oncorhynchus tshawytscha* [15], freshwater cultured shrimp, *M. rosenbergii* [5,6,16], and marine cultured crab, *P. trituberculatus* [4]. An outbreak of *M. bicuspidata* in Taiwan from May 2001 to December 2003 resulted in the cumulative mortality of 20%–95% in *M. rosenbergii* and, in California, *M. bicuspidata* led to the cumulative mortality of 34.5% in larval *O. tshawytscha* [15]. In addition, *M. bicuspidata* occurs in mixed bacterial infections. For example, *M. bicuspidata* was co-infected with *Vibrio alginolyticus* in *M. rosenbergii* [17]. Therefore, this yeast pathogen is easily and widely spread.

Clarifying the transmission route of pathogens is important for the prevention and control of aquaculture disease. *Daphnia magna* infected with *M. bicuspidata* release a large number of ascospores into the environment after death [18]. Healthy *D. magna* can then become infected after ingesting the ascospores [18]. The mature ascospores of *M. bicuspidata* are needle-shaped, allowing them to penetrate their host more easily [19]. In addition, *M. bicuspidata* can spread among different species via the food chain. For example, *M. bicuspidata* infects the brine shrimp *Artemia*, which can act as a vector for transmission to larval salmon [15]. *M. bicuspidata* is a fungal pathogen, and there is no effective drug for treating the infection. Although some antifungal drugs and biological remedies have shown effective anti-pathogenic effects in vitro [8,20], utilization is difficult in aquaculture settings and, more importantly, they are not preferred as the crabs will ultimately be used for human consumption. Therefore, preventing the transmission of *M. bicuspidata* is the best method of control, and research should focus on this aspect. However, it is unclear how healthy crabs are infected. In the present study, healthy crabs were exposed to *M. bicuspidata* in three ways, feeding, immersion, and cohabitation.

## 2. Materials and Methods

### 2.1. Animals

Three hundred and sixty juvenile crabs, weighing 8.2 g ± 0.5 g were obtained from a commercial aquaculture farm in Panjin city, China. To ensure the absence of yeast infection, 20 crabs were randomly selected for microbial and polymerase chain reaction (PCR) testing. The hemolymph samples were streaked onto Rose Bengal agar. The inoculated plates were incubated at 28 °C for 48 h. The hemolymph samples were used for DNA extraction and PCR assay; primers and PCR procedures are described in Section 2.5. No yeast growth was observed on the Rose Bengal medium, and the PCR test was negative. The healthy crabs were acclimated in three 300 L tanks (120 crabs per tank) for one week under continuous aeration and were fed twice a day with a commercial pelleted feed (Wellhope Aquatic Feed Co. Ltd., Shenyang, China). During the acclimation period, the water was maintained at 20 ± 1 °C, pH 7.2 ± 0.2, O_2_ > 5 mg/L, ammonia nitrogen < 0.1 mg/L, nitrite nitrogen < 0.05 mg/L, and waste was removed daily from each tank. For the horizontal transmission experiment, 210 crabs were randomly selected and were divided into six groups.

The diseased crabs used for the cohabitation exposure were collected from a rice field used for crab culture in Panjin; 20 of them were randomly selected for detection of the infection by PCR testing [11].

Hemolymph was collected from 10 diseased berried crabs obtained from a berried crab overwintering pond in Panjin, and the crabs were cultured in 300 L tanks. After hatching, the adult crabs were removed in a timely manner, and the larvae continued to be cultivated in the original container until megalopae developed.

### 2.2. Yeast Stains

The yeast strain *M. bicuspidata* LNES0119 was isolated from a clinically diagnosed, milky diseased *E. sinensis* from Panjin. The *M. bicuspidata* yeast strain was grown in a Rose Bengal medium at 28 °C for 30 h. The cells in the culture were collected and washed with sterile phosphate-buffered saline (PBS) solution, followed by centrifugation at 5000 rpm for 10 min. The pellet obtained was suspended in a sterile PBS solution and the cell density was adjusted to 10^10^ cells/mL as stock solution.

### 2.3. Horizontal Transmission

The experimental transmission was performed in three challenges: feeding challenge (Group I), immersion challenge (Group II), and cohabitation challenge (Group III). For each challenge, control and infected groups were established. All experiments were carried out in triplicate.

For Group I, 10 crabs were transferred into one aquarium and were fed with chopped fresh hepatopancreas, muscle, and gill tissue of yeast-infected crabs twice per day for 3 d. The control group was fed with uninfected tissue. All water was changed daily. After 3 d of culture, feeding of the yeast-infected tissue was discontinued and replaced by commercial pellet feed, fed twice daily.

For Group II, 30 crabs were immersed in water at a yeast concentration of 10^7^ cells/mL. Another 30 crabs were used as a control and were maintained under normal conditions, without exposure to the yeast. Ten crabs were held in one aquarium (50 cm × 30 cm × 30 cm, containing 5 L of water). During the culture period, one-third of the water was changed every day, and the concentration of yeast in the water was maintained at the same level. After 3 d of immersion, the crabs were transferred to water without yeast, as with the control group, for subsequent culture (30 L water).

For Group III, three containers, each containing 30 L of fresh water, were prepared with 10 infected and 10 healthy juvenile crabs per aquarium, separated by a plastic net to prevent physical contact. A negative control group was also established with 10 healthy crabs inside basket cages and 10 crabs outside, as described above. If a diseased crab died, it was quickly removed and replaced with a new diseased crab. One-third of the water was changed daily. After 3 d of culture, the diseased crabs and basket cages were removed from the aquarium.

Gross signs of disease and mortality of the crabs were recorded daily, and dead crabs were removed upon observation. After 35 d, all surviving crabs were dissected after being anesthetized on ice for 5 min. The hepatopancreas and muscle tissues were frozen at −80 °C for later PCR testing. Crabs exhibiting typical clinical signs of infection were dissected. Yeast samples were collected from the hemolymph, re-isolated, and identified by PCR, as described in Section 2.5. The tissues were also fixed with 4% paraformaldehyde for 24 h and then transferred to a 70% alcohol solution for histological examination.

### 2.4. Vertical Transmission Detection

First, ovary tissue from diseased crabs and healthy crabs was fixed with 4% paraformaldehyde for histological examination. Second, 100 fertilized eggs were separately collected from 10 berried crabs that were determined to be infected by PCR analysis of the hemolymph, and the eggs were tested for infection using PCR. Then, these berried crabs were hatched in 300 L tanks until the stage of larval development into megalopae. During this period, zoeae 1–5 and megalopae were collected to detect the presence of *M. bicuspidata*. In addition, zoeae 1–5 and megalopae in the seedling production pond were collected to detect the presence of *M. bicuspidata*. The infection rate of the berried crabs in this pond was 30%. Because crab larvae were small, twenty larvae (zoea 1, zoea 2, zoea 3, zoea 4, zoea 5, and megalopae) were respectively mixed into one sample for DNA extraction. Whether cultured in tanks or in the seedling pond, the larvae were tested three times at each developmental stage.

### 2.5. DNA Extraction and PCR Assay

DNA was extracted using a Tissues Genomic DNA Isolation Kit (Tiangen) according to the manufacturer’s instructions. The primers were designed using a D1/D2 domain of 26S rDNA, as previously reported by Bao et al. [11]. The amplification primers were NL-1 (5′-GCATATCAATAAGCGGAGGAAAAG-3′) and NL-4 (5′-GGTCCGTGTTTCAAGACGG-3′). The PCR protocol was performed as follows: denaturation at 94 °C for 5 min, 35 cycles of denaturation at 94 °C for 30 s, annealing at 52 °C for 1 min, extension at 72 °C for 1 min, and a final extension at 72 °C for 5 min. The PCR product was sequenced by Sangon Biotech (Shanghai, China) for verification of the presence of *M. bicuspidata.*

### 2.6. Histological Section Observation and Smear Examination

Muscle, hepatopancreas, heart, and gill tissues from moribund crabs were immersion-fixed with 4% paraformaldehyde and stained using the standard hematoxylin and eosin staining method [11]. Tissue sections were then examined with a BX41 microscope (Olympus, Japan) for the histological presence of infection. Simultaneously, 2 μL of hemolymph was drawn for smear examination. The smear slide was dried at 20 °C and was then visualized under light microscopy after 2% *w/v* Phloxin B staining.

### 2.7. Statistical Analyses

The Kaplan–Meier survival curves for the different groups were plotted in GraphPad Prism (Version 5.01 for Windows, San Diego, CA, USA) and differences in survival were tested for significance using the log-rank (Mantel–Cox) test. A one-way ANOVA using SPSS 20.0 software was performed to compare the differences in cumulative mortality and infection rate, followed by Tukey’s multiple comparison tests. The level of statistical significance was set at *p* < 0.05.

## 3. Results

### 3.1. Horizontal Transmission

**Figure 1:**
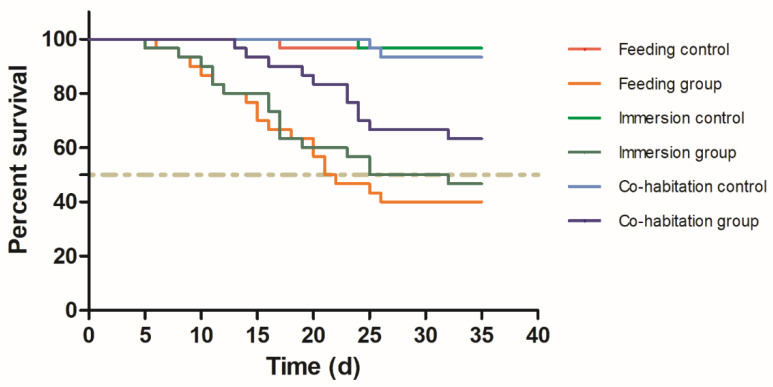
The Kaplan–Meier survival curves of *Eriocheir sinensis* challenged with *Metschnikowia bicuspidata* by feeding, immersion, and cohabitation.

**Figure 2:**
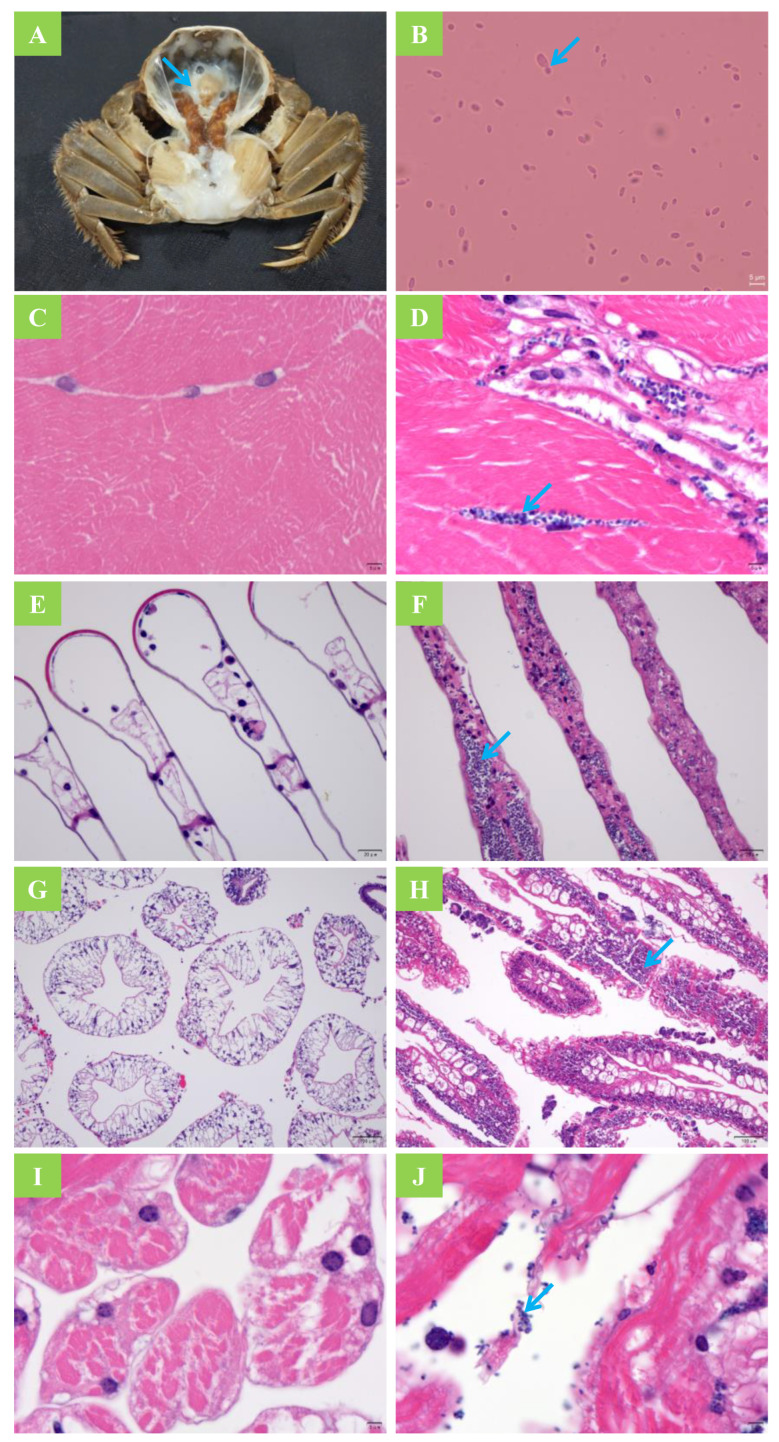
Symptoms and tissue sections after *Metschnikowia bicuspidata* infection. (**A**) Infected crab symptoms; the arrow shows typical milky body fluid. (**B**) A tissue smear of an infected crab; the arrow indicates budding (1000×). (**C**,**D**) A muscle tissue section of uninfected and infected crabs, respectively; the arrow indicates an accumulation of yeast in a broken fiber gap (1000×). (**E**,**F**) A gill tissue section of uninfected and infected crabs, respectively; the arrow indicates yeast within the branchial lamellar vasculature (400×). (**G**,**H**) A hepatopancreas tissue section of uninfected and infected crabs, respectively; the arrow indicates an accumulation of yeast in the hepatopancreatic tubules (100×). (**I**,**J**) A heart tissue section of uninfected and infected crabs, respectively; the arrow indicates an accumulation of yeast in the broken myocardial fibers (1000×).

The survival rate was lower (*p* ˂ 0.05) in all challenge groups compared to the control group (Figure 1). In addition, the cumulative mortality of crabs in the feeding challenge experiment was higher than that in the cohabitation challenge (*p* < 0.05). After 35 d, the infection rate of the control group was 0; however, all three exposure groups exhibited relatively high infection rates (Table 1). The results indicate that healthy crabs can be successfully infected by feeding them infected tissue, immersion in yeast-rich water, or cohabitation with yeast-infected crabs. The PCR analysis of pure cultured yeasts re-isolated from infected crabs also identified *M. bicuspidata*. After being fed yeast-infected tissue, healthy crabs began to die at day 6, and the highest number of deaths occurred from 15 to 21 d. The mortality rate was 60% and the infection rate was 76.7% at 35 d. In the immersion group, the mortality rate was 53.3% and the infection rate was 66.7% at 35 d. The mortality and infection rates of the cohabitation group were lower than those of the feeding group (*p* < 0.05). In addition, the initial mortality in the cohabitation group occurred later than in the feeding and immersion groups. The dead crabs generally had typical symptoms of milky disease including severe hemolymph emulsification and white coloration of the gill, heart, skeletal muscle, and other tissues (Figure 2A). An increased abundance of yeast with budding was observed in the hemolymph smears (Figure 2B). The muscle fibers of the healthy crabs were closely arranged and showed an obvious banded structure (Figure 2C). The muscle fibers of the diseased crabs were loosely arranged and broken. There was a large amount of *M. bicuspidata* invasion in the broken fiber gaps (Figure 2D). The gill lamellae of the healthy crabs had a reticular transverse septum structure composed of epithelial cells (Figure 2E). Whereas the branchial lamellar vasculature of the diseased crabs accumulated a large number of *M. bicuspidata*, resulting in damage to the transverse septum structure, the expansion and deformation of the gill lamellae lumen, and the degeneration and necrosis of epithelial tissue (Figure 2F). The hepatopancreas of the healthy crabs was composed of hepatic tubules with many branches, and the basement membrane and lumen were intact (Figure 2G). The hepatopancreatic tubules of the diseased crabs were arranged irregularly, the basement membrane was broken, the structure of the hepatocytes was disintegrated, and the hepatopancreatic tubules were filled with an abundance of *M. bicuspidata* (Figure 2H). The myocardial fibers of the healthy crabs were arranged neatly, and the nucleus was oval and located at the edge of the cell (Figure 2I). Due to the invasion of *M. bicuspidata*, the myocardial fibers of the diseased crabs were broken and necrotic (Figure 2J).

### 3.2. Vertical Transmission

**Figure 3:**
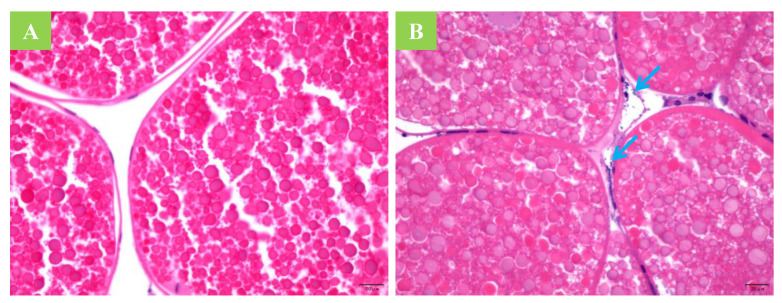
Ovarian tissue sections of a healthy crab and of a crab after *Metschnikowia bicuspidata* infection. (**A**) A healthy crab (400×). (**B**) A diseased crab; the arrow indicates that *Metschnikowia* *bicuspidata* can enter the ovarian tissue, but not the oocyte (400×).

A histological examination of the ovarian tissue from the infected crabs, as determined by PCR analysis of the hemolymph, did not reveal intra-oocyte yeast (Figure 3). Likewise, near-term fertilized eggs, zoeae in stages 1–5, and megalopae from the infected crabs tested negative for the yeast by PCR, indicating that vertical transmission did not occur. Similarly, *M. bicuspidata* was not detected by PCR testing during the larval stage of hatching in the pond.

## 4. Discussion

*Metschnikowiabicuspidata* is a pathogenic yeast that was first identified in *Daphnia* [21]. This pathogen can infect various economically important aquatic animals, such as *P. trituberculatus*, *M. rosenbergii*, and *E. sinensis*, which results in substantial economic losses to the aquaculture industry [8,16,22]. Therefore, it is important to identify the route of transmission to effectively control mycoses in these cultured species. Previous research has shown that salmon can also be infected with *M. bicuspidata*, via *Artemia* as the vector, but it cannot be infected directly from the water or through intramuscular injection [15]. Unlike vertebrates, crustaceans have an open tube cycle. Hemolymph not only flows through the heart and blood vessels but also into the cell space. Therefore, *M. bicuspidata* can be rapidly infected by injection. This has been confirmed in *P. trituberculatus*, *M. rosenbergii*, and *E. sinensis* [4,11,12,16]. However, to the best of our knowledge, there have been no studies that discuss intra-species or inter-species transmission.

In general, aquatic animal pathogens are transmitted via horizontal transmission, vertical transmission, or both [23,24,25]. The route of transmission is related to the type of pathogen. Generally, pathogens that are spread through vertical transmission have low virulence, which keeps the host alive, while those that are spread through horizontal transmission are often highly virulent, which accelerates the diffusion rate following the death of the host [26,27,28]. Fungi are mainly spread through horizontal transmission.

In the present study, we found that *M. bicuspidata* did not colonize germ cells and was not detected in fertilized eggs and hatched larvae (zoeae 1–5 and megalopae), which indicated that *M. bicuspidata* was not transmitted vertically. Similarly, the prevalence of *M. bicuspidata* in *M. rosenbergii* was highest in adult prawns (73%), followed by juveniles (25%) and post-larvae (2%), but no yeast infections were detected in larvae [5]. Owing to the metamorphosis and rapid development of the larval stage, an infection experiment of the larval stage was not included in this investigation, and further studies are required to determine whether infection with *M. bicuspidata* is possible during the metamorphosis stage. A previous investigation demonstrated that the infection rate in young crabs was low, but the infection rate in the adult stage was high [10], which is consistent with the observations in *M. rosenbergii* [16], although the reason for the high rate of infections in adults remains unclear. Chen et al. [16] postulated that despite adults having a more mature immune defense system, they are more likely than young shrimp to encounter yeast in pond water or sediment because they have a longer life span and tend to consume dead or dying shrimp that have been infected with the yeast.

Similar to other fungi, *M. bicuspidata* is mainly transmitted through horizontal transmission. In the present study, healthy crabs were infected with the yeast following the ingestion of infected tissues, indicating that cannibalism is a mode of infection. The *Eriocheir* *sinensis* is aggressive and often cannibalizes other crabs, especially when the shell has not hardened after molting [29,30]. Pathogen transmission through mutual encroachment is also common in crustaceans, such as with the white spot syndrome virus (WSSV) in *P. vannamei* [31]. In high-density breeding conditions, it is difficult to avoid cannibalism, and disease-carrying organisms are easy to consume given their weakened state, which further facilitates the transmission of pathogens. Co-cultured carnivorous fish can significantly reduce the incidence of WSSV in *P. vannamei* [32] because carnivorous fish rapidly consume moribund shrimp, which prevents transmission of the pathogen to healthy shrimp. However, it is difficult to use this method in crab cultures, as it is difficult for common fish to consume the hard, thickened shell of crabs, and paddy field environments are also not conducive to the polyculture of fish.

Soto et al. [33] compared the transmission rate of WSSV in *P. vannamei* through feeding and by cohabitation. Their results showed that the transmission rate of WSSV through cohabitation was 0.01, while that from feeding was 0.46, which indicates that cohabitation is not a major route of transmission for WSSV. The present study demonstrated that healthy crabs can still be infected when they are not in contact with diseased crabs and during mixed breeding, healthy crabs can become infected by the ingestion of feces or water containing the pathogen. In addition, this investigation revealed that healthy crabs can become infected when exposed to water contaminated with *M. bicuspidata*. Owing to the high concentration of *M. bicuspidata* (10^7^ cells/mL) used in the experiment, the infection rate from immersion was also high, but such a concentration is unusual during the culture process. Therefore, the minimal concentration of yeast in the culture environment requires further investigation. Previous research has shown that yeast mainly infects aquatic organisms via wounds caused by aggression or mechanical injury [34,35,36], although the present study demonstrated that healthy crabs without external lesions can be infected by yeast, which is consistent with the findings of Stentiford et al. [37]. Lu et al. [5] also suggested that *M. rosenbergii* infection occurs mainly through mouth and gill contact with the yeast in water and sediments.

Through comparing the three modes of infection, the infection rate of the group that was fed infected tissue was higher than the bath immersion and cohabitation groups, suggesting that cannibalism is more likely to result in infection. The survey of juvenile crabs and berried crabs in the overwintering ponds in the Panjin area also revealed that the infection rate was high [10]. This may be related to the high-density culture under the ice during the overwintering period, without access to shelter, which results in the cannibalism of infected crabs. This study also revealed that feeding, bath immersion, and cohabitation did not reach a 100% infection rate, further suggesting that there were individuals with a strong resistance to *M. bicuspidata* in the population, which highlights a promising avenue for the future breeding of disease-resistant *Eriocheir* *sinensis* and other species.

Many studies have been carried out with respect to the treatment of fungal diseases. Ma et al. [12,38] found that common antifungal drugs (polyenes, triazoles, and fluorocytosine) markedly inhibited *M. bicuspidata* in vitro. Zhang et al. [20], found that massoia lactone, derived from liamocins produced by *Aureobasidium melanogenum*, performed well against *M. bicuspidata*. However, the absorption, distribution, metabolic kinetics, and associated by-products of these antifungal treatments have not been well investigated. Considering factors such as food safety and production cost, it is unclear whether these treatments can be used safely on a large scale. Some researchers have tried to control the disease with biological approaches, including using marine killer yeast against *M. bicuspidata* [8,39,40,41]. Although these killer yeasts can inhibit *M. bicuspidata* in a laboratory environment, it is unclear how they may safely be used in complex aquacultur environments and achieve a significant effect. It is expected that safe and effective drugs against yeast infections will be developed in the near future. Until that time, prevention is the best strategy. Furthermore, since transmission of the pathogen can occur via contaminated water and infected tissues, as demonstrated in the present study, additional measures to reduce infection include disinfecting the water and bait organisms, reducing the culture density, and removing dead individuals.

## 5. Conclusions

*Metschnikowia bicuspidata* was not shown to infect gonadal cells or fertilized egg embryos in this study, suggesting that *M. bicuspidata* is not vertically transmitted. However, infection experiments demonstrated that *M. bicuspidata* can be transmitted to healthy crabs by feeding them yeast-infected tissue, immersion in contaminated water, or through cohabitation with infected crabs. Clarifying these transmission routes is necessary for the prevention of *M. bicuspidata* infection in *E. sinensis*.

## Figures and Tables

**Table 1:** The cumulative mortality and infection rate of different infection modes.

	Crab Number	Number of Deaths at Different Times					Cumulative Mortality (%)	Infection Rate (%)
		1–7 d	8–14 d	15–21 d	22–28 d	29–35 d		
Feeding control	30	0	0	1	0	0	3.3 ± 5.8 ^C^	0 ^C^
Feeding group	30	1	6	8	3	0	60 ± 10 ^A^	76.7 ± 10 ^A^
Immersion control	30	0	0	0	1	0	3.3 ± 5.8 ^C^	0 ^C^
Immersion group	30	1	5	6	3	1	53.3 ± 11.5 ^AB^	66.7 ± 5.8 ^AB^
Cohabitation control	30	0	0	0	2	0	6.7 ± 5.8 ^C^	0 ^C^
Cohabitation group	30	0	2	3	5	1	36.7 ± 5.8 ^B^	53.3 ± 11.5 ^B^

Notes: different letters in the same column indicate significant differences.

## Data Availability

All data included in this study are available upon request from the corresponding author.

## References

[1] Stentiford G.D. (2008). Diseases of the European edible crab (*Cancer pagurus*): A review. J. Mar. Sci..

[2] Guerra R.S., do Nascimento M.M.F., Miesch S., Najafzadeh M.J., Ribeiro R.O., Ostrensky A., de Hoog S., Vicente V., Boeger W.A. (2013). Black yeast biota in the mangrove, in search of the origin of the lethargic crab disease (LCD). Mycopathologia.

[3] Cai W.Q. (1996). A study on pathology of the disease caused by *Torulopsis Mogii* in giant freshwater prawn *Macrobrachium rosenbergii*. J. Fish. China.

[4] Xu W., Xu H., Shi H., Qian D. (2005). Preliminary study on *Candida oleophila* disease in *Portunus trituberculatus*. J. Fish. China.

[5] Lu C.C., Tang K.F.J., Chen S.N. (1998). Identification and genetic characterization of yeasts isolated from freshwater prawns, *Macrobrachium rosenbergii* de man, in Taiwan. J. Fish Dis..

[6] Chen S.C., Chen T.H., Wang P.C., Chen Y.C., Liaw L.L. (2003). *Metschnikowia bicuspidate* and *Enterococcus faecium* coinfection in the giant freshwater prawn *Macrobrachium rosenbergii*. Dis. Aquat. Org..

[7] Vicente V.A., Orélis-Ribeiro R., Najafzadeh M.J., Sun J.F., Guerra R.S., Miesch S., Ostrensky A., Meis J.F., Klaassen C.H., Hoog G.S. (2012). Black yeastlike fungi associated with lethargic crab disease (LCD) in the mangrove-land crab, *Ucides cordatus* (Ocypodidae). Vet. Microbiol..

[8] Wang X., Chi Z., Yue L., Li J., Li M., Wu L. (2007). A marine killer yeast against the pathogenic yeast strain in crab (*Portunus trituberculatus*) and an optimization of the toxin production. Microbiol. Res..

[9] Fishery Administration of the Ministry of Agriculture and Rural Areas (2020). China Fishery Statistical Yearbook.

[10] Sun N., Bao J., Liang F., Liu F., Jiang H., Li X. (2022). Prevalence of ‘milky disease’ caused by *Metschnikowia bicuspidata* in *Eriocheir sinensis* in Panjin city, China. Aquac. Res..

[11] Bao J., Jiang H., Shen H., Xing Y., Feng C., Li X., Chen Q. (2021). First description of milky disease in the Chinese mitten crab *Eriocheir sinensis* caused by the yeast *Metschnikowia bicuspidata*. Aquaculture.

[12] Ma H., Lu X., Liu J., Guo S., Zhao X., Ye S. (2021). *Metschnikowia bicuspidata* isolated from milky diseased *Eriocheir sinensis*: Phenotypic and genetic characterization, antifungal susceptibility and challenge models. J. Fish Dis..

[13] Codreanu R., Codreanu-Balcescu D. (1981). On two Metschnikowia yeast species producing hemocoelic infections in *Daphnia magna* and *Artemia salina* (Crustacea, Phyllopoda) from Romania. J. Invertebr. Pathol..

[14] Shaw C.L., Bilich R., O’Brien B., Cáceres C.E., Hall S.R., James T.Y., Duffy M.A. (2021). Genotypic variation in an ecologically important parasite is associated with host species, lake, and spore size. Parasitology.

[15] Moore M.M., Strom M.S. (2003). Infection and mortality by the yeast *Metschnikowia bicuspidata* var. bicuspidata in chinook salmon fed live adult brine shrimp (Artemia franciscana). Aquaculture.

[16] Chen S.C., Chen Y.C., Kwang J., Manopo I., Wang P.C., Chaung H.C., Liaw L.L., Chiu S.H. (2007). *Metschnikowia bicuspidata* dominates in Taiwanese cold-weather yeast infections of *Macrobrachium rosenbergii*. Dis. Aquat. Org..

[17] Wang G.L., Shan J., Chen Y.E., Li Z. (2006). Study on pathogens and pathogenesis of emulsification disease of *Portunus trituberculatus*. Adv. Mar. Sci..

[18] Merrill T.E.S., Cáceres C.E. (2018). Within-host complexity of a plankton-parasite interaction. Ecology.

[19] Lachance M.A., Miranda M., Miller M.W., Phaff H.J. (1976). Dehiscence and active spore release in pathogenic strains of the yeast *Metschnikowia bicuspidata* var. australis: Possible predatory implication. Can. J. Microbiol..

[20] Zhang H.Q., Chi Z., Liu G.L., Zhang M., Hu Z., Chi Z.M. (2021). *Metschnikowia bicuspidate* associated with a milky disease in *Eriocheir sinensis* and its effective treatment by *Massoia lactone*. Microbiol. Res..

[21] Metschnikoff E. (1884). A disease of Daphnia caused by a yeast. A contribution to the theory of phagocytes as agents for attack on disease-causing organisms. Archiv. Pathol. Anat. Physiol. Klin. Med..

[22] Jiang H., Bao J., Xing Y., Feng C., Li X., Chen Q. (2021). Proteomic Analysis of the Hemolymph after *Metschnikowia bicuspidata* Infection in the Chinese Mitten Crab *Eriocheir sinensis*. Front. Immunol..

[23] Bright M., Bulgheresi S. (2010). A complex journey: Transmission of microbial symbionts. Nat. Rev. Microbiol..

[24] Stentiford G.D., Feist S.W., Stone D.M., Bateman K.S., Dunn A.M. (2013). Microsporidia: Diverse, dynamic, and emergent pathogens in aquatic systems. Trends Parasitol..

[25] Svoboda J., Mrugała A., Kozubíková-Balcarová E., Petrusek A. (2017). Hosts and transmission of the crayfish plague pathogen *Aphanomyces astaci*: A review. J. Fish Dis..

[26] Lipsitch M., Siller S., Nowak M.A. (1996). The evolution of virulence in pathogens with vertical and horizontal transmission. Evolution.

[27] Alizon S. (2013). Parasite co-transmission and the evolutionary epidemiology of virulence. Evol. Int. J. Org. Evol..

[28] Pagán I., Montes N., Milgroom M.G., García-Arenal F. (2014). Vertical transmission selects for reduced virulence in a plant virus and for increased resistance in the host. PLoS Pathog..

[29] Yang X.Z., Pang Y.Y., Huang G.Y., Xu M.J., Zhang C., He L., Lv J.H., Song Y.M., Song X.Z., Cheng Y.X. (2019). The serotonin or dopamine by cyclic adenosine monophosphate-protein kinase a pathway involved in the agonistic behaviour of Chinese mitten crab, *Eriocheir sinensis*. Physiol. Behav..

[30] Li Y., Jiang Q., Fan S., Sun N., Li X.D., Zheng Y. (2019). Aggressive behavior variation and experience effects in three families of juvenile Chinese mitten crab (*Eriocheir sinensis*). Behav. Processes.

[31] Lotz J.M., Soto M.A. (2002). Model of white spot syndrome virus (WSSV) epidemics in *Litopenaeus vannamei*. Dis. Aquat. Org..

[32] Dey B.K., Dugassa G.H., Hinzano S.M., Bossier P. (2020). Causative agent, diagnosis and management of white spot disease in shrimp: A review. Rev. Aquac..

[33] Soto M.A., Lotz J.M. (2001). Epidemiological parameters of white spot syndrome virus infections in *Litopenaeus vannamei* and *L. setiferus*. J. Invertebr. Pathol..

[34] Hussain M., Summerfelt R.C. (1991). The role of mechanical injury in an experimental transmission of *Flexibacter columnaris* to fingerling walleye. J. Iowa Acad. Sci. JIAS.

[35] Bader J.A., Nusbaum K.E., Shoemaker C.A. (2003). Comparative challenge model of *Flavobacterium columnare* using abraded and unabraded channel catfish, *Ictalurus punctatus* (Rafinesque). J. Fish Dis..

[36] Bader J.A., Moore S.A., Nusbaum K.E. (2006). The effect of cutaneous injury on a reproducible immersion challenge model for *Flavobacterium columnare* infection in channel catfish (*Ictalurus punctatus*). Aquaculture.

[37] Stentiford G.D., Evans M., Bateman K., Feist S.W. (2003). Co-infection by a yeast-like organism in Hematodinium-infected European edible crabs *Cancer pagurus* and velvet swimming crabs *Necora puber* from the English Channel. Dis. Aquat. Org..

[38] Ma H., Sun N., Lu X., Liu J., Guo S., Zhao X., Ye S. (2020). Isolation and identification of pathogen in milky disease of Chinese mitten crab *Eriocheir sinensis* in Liaoning Province. J. Dalian Ocean. Univ..

[39] Wang L., Yue L., Chi Z., Wang X. (2008). Marine killer yeasts active against a yeast strain pathogenic to crab *Portunus trituberculatus*. Dis. Aquat. Org..

[40] Buzdar M.A., Chi Z., Wang Q., Hua M.X., Chi Z.M. (2011). Production, purification, and characterization of a novel killer toxin from *Kluyveromyces siamensis* against a pathogenic yeast in crab. Appl. Microbiol. Biotechnol..

[41] Tan C., Wang L., Xue Y., Yu G., Yang S., Lin S. (2018). Marine killer yeast *Metschnikowia saccharicola* active against pathogenic yeast in crab and an optimization of the toxin production. Afr. J. Biotechnol..

